# miR-139-5p Inhibits the Epithelial-Mesenchymal Transition and Enhances the Chemotherapeutic Sensitivity of Colorectal Cancer Cells by Downregulating BCL2

**DOI:** 10.1038/srep27157

**Published:** 2016-05-31

**Authors:** Qingguo Li, Xin Liang, Yuwei Wang, Xianke Meng, Ye Xu, Sanjun Cai, Zhimin Wang, Jianwen Liu, Guoxiang Cai

**Affiliations:** 1 Department of Colorectal Surgery, Fudan University Shanghai Cancer Center, Shanghai 200032, China; 2 Department of Oncology, Shanghai Medical College, Fudan University, Shanghai 200032, China; 3 State Key Laboratory of Bioreactor Engineering & Shanghai Key Laboratory of New Drug Design, School of Pharmacy, East China University of Science and Technology, Shanghai 200237, China; 4 Department of Genetics, Shanghai-MOST Key Laboratory of Health and Disease Genomics, Chinese National Human Genome Center and Shanghai Academy of Science & Technology, Shanghai 201203, China

## Abstract

MicroRNAs (miRNAs) are important regulators involved in various cancers, including colorectal cancer (CRC). The functions and mechanisms of the miRNAs involved in CRC progress and metastasis are largely unknown. In this study, miRNA microarray analysis was performed to screen crucial miRNAs involved in CRC progress, and miR-139-5p was chosen for further study. The functional roles of miR-139-5p in colon cancer were demonstrated by CCK-8 proliferation assay, cell invasion and migration, cell apoptosis and in a KO mouse study. miR-139-5p expression was significantly decreased in cancer tissues compared to normal tissues. The miR-139-5p expression level was associated with tumour stage (*P* < 0.01). Function studies revealed that miR-139-5p was significantly correlated with the metastasis potential and drug resistance of colon cancer cells by affecting the epithelial-mesenchymal transition (EMT). Then, we identified BCL2 as a direct target of miR-139-5p cells *in vitro*. The patient samples and KO mice model showed that BCL2 expression was inversely correlated with the expression of miR-139-5p. In conclusion, we found that miR-139-5p targeted the BCL2 pathway to reduce tumour metastasis and drug sensitivity in CRC. This axis provided insight into the mechanism underlying miRNA regulation of CRC metastasis and a novel therapeutic target for CRC therapy.

Colorectal cancer is the third most common cancer and the second leading cause of cancer death in women and men worldwide^1^. The development of colorectal cancer involves multiple factors, including inactivation of tumour suppressor genes and activation of oncogenes. Improved understanding of the tumour biology and mechanism may enhance surgical strategies, chemotherapy methods, and follow-up strategies. During the past decade, a type of non-protein-coding RNA molecule known as microRNA (miRNA) has been linked to tumourigenicity and tumour progression by acting as either tumour suppressors or oncogenes^2^,^3^. miRNAs are post-transcriptional regulators of gene expression that bind primarily to complementary sequences in the 3′ untranslated region (UTR) of their target mRNAs and cause translational repression or mRNA degradation of target genes^2^,^3^,^4^. miRNAs are estimated to regulate more than one-third of human genes and to influence their function and genetic pathways^5^. Increasing evidence suggests that aberrant miRNA expression is involved in the invasion and metastasis of colorectal cancer^2^,^3^,^6^. However, the functions and mechanisms of miRNAs in colorectal cancer metastasis are not fully understood.

In this study, we performed miRNA arrays in six paired colorectal cancer and normal tissues to detect miRNAs involved in invasion and metastasis. miRNAs that are differentially expressed in the six paired tissues may directly regulate the carcinogenesis and progression of colorectal cancer. Among the candidate miRNAs, miR-139-5p was chosen for further study, and we found miR-139-5p was often downregulated in colorectal cancer. Importantly, miR-139-5p was negatively associated with metastasis and drug resistance and could be an independent prognostic factor for colorectal cancer patients. Mechanistically, our study demonstrated that BCL2 is a direct and functional target for miRNA-139-5p in colorectal cancer.

## Results

### miR-139-5p was downregulated in colon cancer cell lines and patient specimens

**Figure 1:**
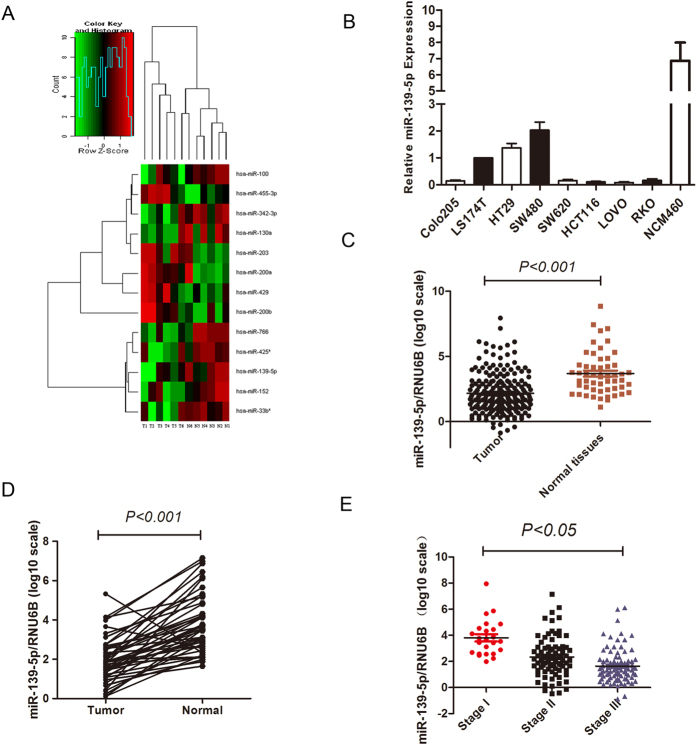
miR-139-5p was downregulated in colorectal cancer. (**A**) Heat map. miRNA microarray analyses were performed in six paired tumour and normal tissues. The green in the legend represents downregulation, and the red represents upregulation. (**B**) Determination of miR-139-5p expression levels in eight colon cell lines and NCM460 cells (mean** ± **SEM). (**C,D**) Downregulation of miR-139-5p expression in primary colorectal cancer tissue compared their normal tissue (paired Student’s t-test). (**E**) miR-139-5p expression levels decreased significantly with the advance of the tumour stage.

To determine the roles of miRNAs in colorectal cancer, we performed miRNA microarrays (Agilent miRNA microarrays, version 12.0, Santa Clara, CA, USA) in six paired specimens of cancerous and normal colorectal tissue. Among the differentially expressed miRNAs, 12 miRNAs were upregulated or downregulated in the cancer tissues (Fig. 1A). miR-203 and miR-139-5p were the most significantly upregulated or downregulated. miR-203 has been fully studied in colorectal cancers^7^,^8^,^9^,^10^, so we selected miR-139-5p for further study.

Real-time PCR analyses revealed that all eight colon cancer cells, including HT29, LS174T, SW480, SW620, RKO, HCT116, COLO205, and LoVo, express lower miR-139-5p levels than the normal colon cell NCM460. Interestingly, the miR-139-5p expression levels are consistent with the invasive ability of the eight cancer cells (Fig. 1B). Moreover, clinical significance analysis of patient samples showed that miR-139-5p expression levels were lower in cancer tissues than normal colorectal tissues (Fig. 1C,D). Specifically, miR-139-5p expression levels decreased significantly with the advance of the tumour stage (Fig. 1E). Collectively, miR-139-5p expression decreased stepwise in colon cancer cells with gradually increasing metastatic potential and was downregulated in colorectal cancer samples, indicating that miR-139-5p acts as a metastasis suppressor in colorectal cancer.

### miR-139-5p inhibits CRC tumourigenesis and metastasis

**Figure 2:**
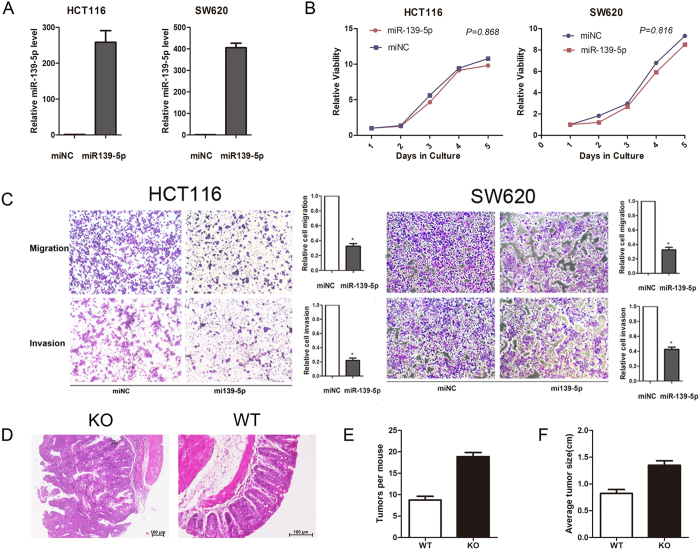
miR-139-5p suppressed colon cancer cells metastasis *in vitro* and *in vivo*. (**A**) The efficiency of miR-139-5p overexpression in colon cancer cell lines was measured by RT-PCR. (**B**) The influence of miR-139-5p expression on the proliferation of colon cancer cells was measured by CCK-8 assay. (**C,D**) Representative results of the migration and Transwell assays showing the effect of miR-139-5p expression on the migratory abilities of HCT116 and SW620 cells (unpaired Student’s t-test, mean** ± **SEM; *P < 0.05). (**E**) Representative images of tumour differentiation in miR-139-5p KO mice and WT mice. KO mice exhibited poorer tumour differentiation than WT mice. (**F**) KO mice showed an approximately twofold increase in tumour numbers compared to their WT littermates (unpaired Student’s t-test, mean** ± **SEM; P < 0.05).

To evaluate the effect of miR-139-5p on HCT116 and SW620 cells, which have low endogenous miR-139-5p expression, we ectopically expressed miR-139-5p in HCT116 and SW620 cells. Real-time PCR was used to verify the increased levels of miR-139-5p in the transfected cells compared with the control cell lines (Fig. 2A). CCK-8 assays were conducted to examine whether miR-139-5p expression affected colon cancer cell proliferation *in vitro*. As shown in Fig. 2B, ectopic miR-139-5p only slightly decreased cell growth *in vitro* (*P* > 0.05). However, the Transwell migration assay and Matrigel invasion assay showed that ectopic expression of miR-139-5p significantly decreased the migration and invasion of HCT116 and SW620 cells (P < 0.05) (Fig. 2C).

In the KO mouse models, we observed earlier tumour formation and poorer tumour differentiation in miR-139-5p KO mice than WT mice (Fig. 2D). KO mice also exhibited an approximately twofold increase in tumour number than their WT littermates (8 *vs* 15) (Fig. 2E). miR-139-5p deficiency also increased the size of tumours (Fig. 2F). These results indicated that miR-139-5p deficiency may enhance colitis-associated tumourigenesis. Collectively, these data suggested that miR-139-5p deficiency not only enhanced tumour development but also increased tumour progression.

### miR-139-5p suppresses the epithelial-mesenchymal transition in human colon adenocarcinoma cells

**Figure 3:**
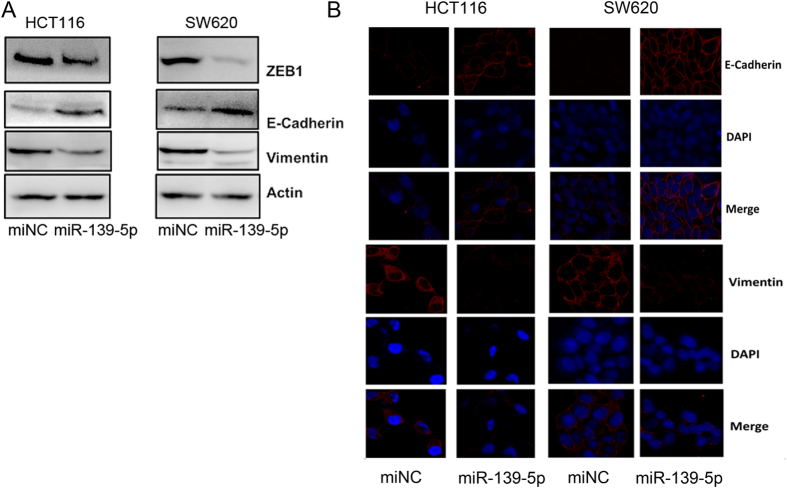
miR-139-5p inhibits the epithelial-mesenchymal transition in colon cancer. (**A**) Western blot analysis of the phenotypic markers, including E-cadherin, Vimentin, and ZEB1 in miR-139-5p overexpression cells. β-Actin expression was used as the loading control. (**B**) Confocal microscopy analysis of the phenotypic markers, including E-cadherin and Vimentin. The red signal represents the staining of the corresponding protein, and the blue signal represents the nuclear DNA staining by DAPI.

Because epithelial-mesenchymal transition (EMT) is closely related to cancer cell metastasis ability, we next examined EMT markers in miR-139-5p transfected and control colorectal cells. Overexpression of miR-139-5p resulted in increased expression of E-cadherin and decreased expression of Vimentin and ZEB1 (Fig. 3A). These results were confirmed by confocal microscopy examination in HCT116 and SW620 cells (Fig. 3B).

### miR-139-5p enhances chemotherapeutic sensitivity in colon cancer cells

**Figure 4:**
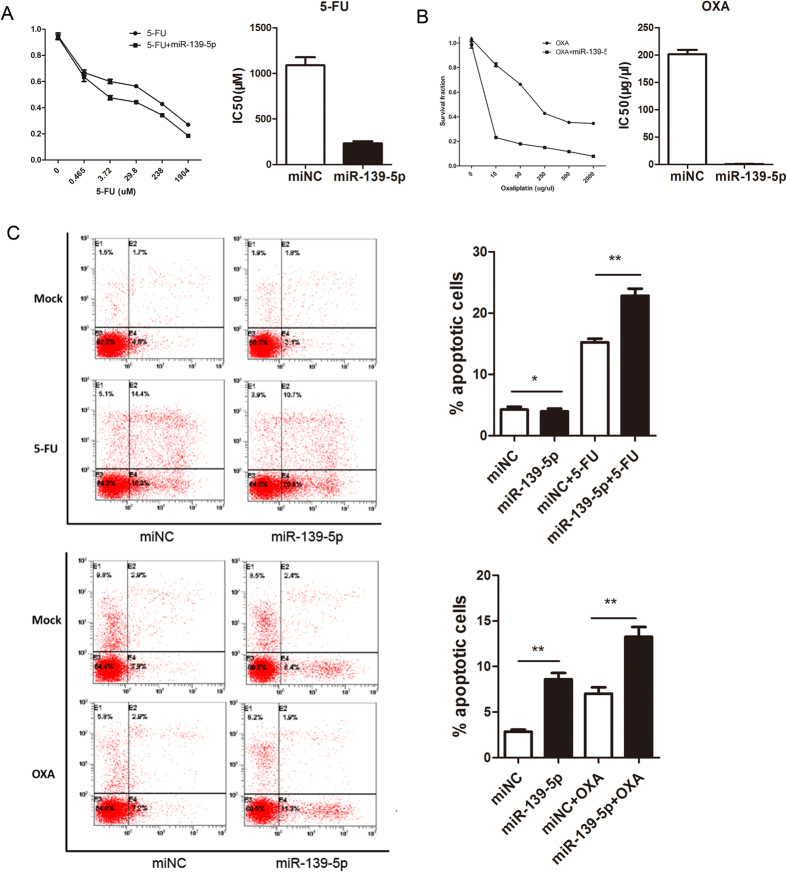
miR-139-5p enhances chemotherapeutic sensitivity in colon cancer cells. (**A,B**) miR-139-5p expression significantly enhanced the sensitivity of HCT116 to 5-FU and OXA and significantly reduced their IC50 based on the CCK-8 assay. (**C**) miR-139-5p-transfected cells showed greater sensitivity towards 5-FU and OXA, with more cells undergoing apoptosis after the treatment (*P > 0.05, **P < 0.05).

The chemotherapy regimens were primarily fluorouracil (5-FU)-based, with leucovorin and oxaliplatin (OXA). To determine the effect of altered miR-139-5p expression on the chemotherapeutic sensitivity of colon adenocarcinoma cells, miR-139-5p-transfected and control stable colon adenocarcinoma cells were used for the assays. Based on the CCK-8 assay, Fig. 4A,B showed that forced miR-139-5p expression significantly enhanced the sensitivity of HCT116 to both 5-FU and OXA and significantly reduced their half maximal inhibitory concentration (IC50). Because induced apoptosis is an important factor of the chemotherapeutic sensitivity of cancer cells, we used flow cytometry to detect the apoptosis capacity of HCT116 miR-139-5p/control cells in the presence of 5-FU or OXA at the IC50. After 24 hour treatment, miR-139-5p-transfected cells showed greater sensitivity towards these chemotherapeutic agents, with more cells undergoing apoptosis after the treatment (P < 0.05) (Fig. 4C). This assay indicated that miR-139-5p is associated with the apoptotic potential.

### BCL2 as a target of miR-139-5p in colorectal cancer cells

**Figure 5:**
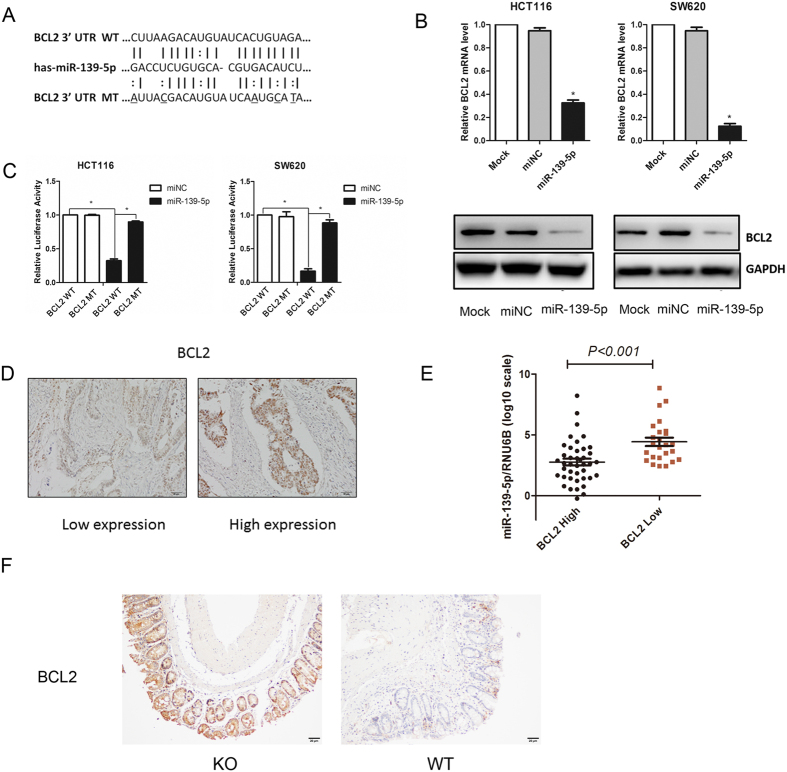
miR-139-5p directly targeted BCL2 in colon cancer cells. (**A**) Schematic representation of the miR-139-5p putative binding sites in the 3′-UTR of BCL2 mRNA and the mutations introduced into the BCL2 3′-UTR regions. (**B**) BCL2 mRNA and protein expression were determined by RT-PCR and Western blotting. (**C**) Wild-type (WT) or mutated (MUT) BCL2 reporter constructs were co-transfected into HCT116 and SW620 cells or controls. The relative luciferase activities were measured. (**D**) Representative images of the IHC staining of BCL2 in colorectal cancer patients. (**E**) miR-139-5p expression levels were inversely correlated with BCL2 expression in colorectal cancer samples. *P < 0.001. (**E**) Representative images of the IHC examination of BCL2 in mouse tumours from miR-139-5p KO and WT mice.

Bioinformatics databases (TargetScan and MicroCosm) identified BCL2 as a potential target of miR-139-5p, and putative binding sites for miR-139-5p in the 3′-UTR of BCL2 were detected (Fig. 5A). The BCL2 oncogene was the first anti-apoptotic gene to be discovered. Based on recent evidence showing that BCL2 is frequently correlated with tumourigenicity^11^, metastasis^12^,^13^, EMT^14^,^15^, decreased susceptibility to chemotherapeutics, and increased radioresistance^16^,^17^, Western blot and RT-PCR analyses were performed, and BCL2 was found to be decreased in miR-139-5p-transfected cells at both the mRNA and protein levels (Fig. 5B).

To investigate the possible interaction between miR-139-5p and BCL2, mutations were introduced into the putative miR-139-5p binding sites in the BCL2 3′-UTR, and luciferase reporter constructs generated with the WT and MUT 3′-UTRs of BCL2 were co-transfected into HCT116 and SW620 cells with the miR-139-5p mimics or miR-control vectors. Luciferase assays showed that ectopic expression of miR-139-5p significantly decreased the luciferase activity of the WT but not that of the MUT BCL2 3′-UTR in both cell lines (Fig. 5C). We further validated the association between miR-139-5p and BCL2 expression levels in 66 colorectal cancer specimens and found high expression of miR-139-5p was always associated with low BCL2 expression. A *t*-test showed that miR-139-5p was inversely correlated with BCL2 expression (P < 0.05) (Fig. 5D,E). miR-139-5p KO mice exhibited stronger BCL2 staining than their control. Collectively, these results suggest that BCL2 is a target of miR-139-5p (Fig. 5F).

### Overexpression of BCL2 decreases the inhibitory effects of miR-139-5p on progression and drug resistance in colorectal cancer cells

**Figure 6:**
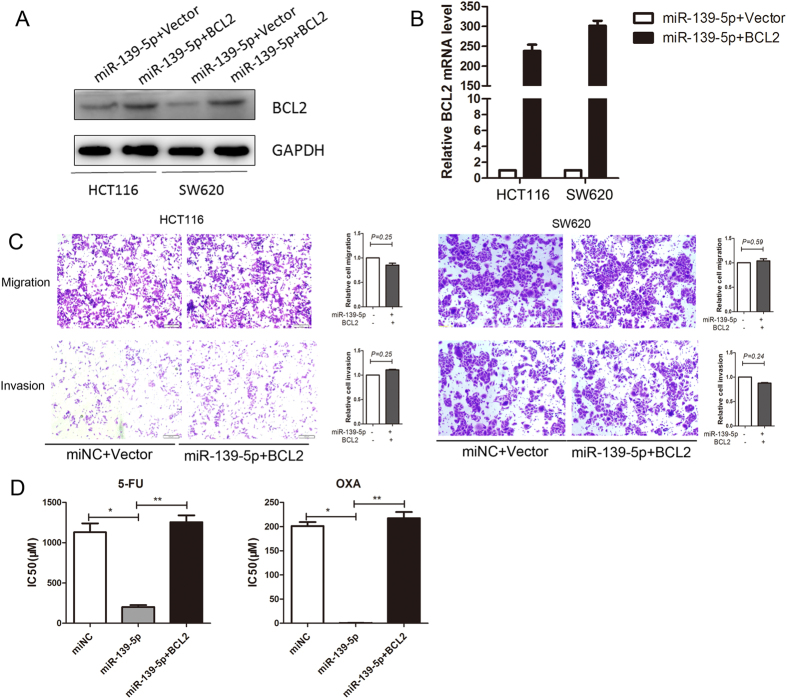
Re-expression of BCL2 abolishes miR-139-5p-induced inhibition of colon cancer metastasis and drug sensitivity. (**A,B**) Western blot and RT-PCR analysis of BCL2 protein expression in HCT116 colon cancer cells expressing miR-139-5p or miR-139-5p plus BCL2. Reintroduction of BCL2 reversed the effects of miR-139-5p on cell migration and invasion (**C,D**) and drug sensitivity (**E**). Each sample was repeated three times. *, **P < 0.05.

To determine whether the role of miR-139-5p in colorectal cancer is mediated by BCL2, we overexpressed BCL2 in miR-139-5p-transfected HCT116 and SW620 cells (Fig. 6A,B). Migration and Transwell assays showed that overexpression of BCL2 fully abolished the suppression of cell migration and invasion induced by miR-139-5p in both HCT116 and SW620 cells (Fig. 6C). Based on CCK-8 assays, we confirmed that overexpression of BCL2 reversed the inhibitory effects of miR-139-5p on drug resistance in colon cancer cells (Fig. 6D). These results confirm that BCL2 is regulated by miR-139-5p, and miR-139-5p downregulation is a factor in colorectal cancer progression and drug resistance through potentiation of BCL2 expression.

## Discussion

The critical role of miRNAs in tumourigenesis via modulation of various targets has promoted intensive research into miRNAs as biomarkers and miRNA-based therapeutic strategies for the treatment of cancer^18^. Despite early diagnoses and advanced surgical strategies, a considerable proportion of colorectal cancer patients develop recurrence or metastasis within 5 years of surgical treatment^19^. To reduce the risk of recurrence and metastasis, 5-FU-based chemotherapy is the standard treatment course for patients with advanced colorectal cancer. However, chemotherapy resistance leading to treatment failure is a critical problem. One of the biggest challenges is to identify the patient subpopulations that are most likely to develop recurrence and metastasis or to respond to specific therapies. If one or more biomarkers could predict these subpopulations, we could more effectively treat these patients.

In the present study, the microRNA expression profile was first examined in colorectal cancer samples. We found that miR-139-5p was frequently downregulated in colorectal cancer patient samples compared to normal colorectal tissues. Subsequent analysis indicated that miR-139-5p was significantly correlated with advanced clinical stage. We then demonstrated that miR-139-5p inhibits EMT and enhances the chemotherapeutic sensitivity of colorectal cancer cells by regulating BCL2 expression *in vitro*. In colorectal cancer samples and miR-139-5p mice, the expression levels of miR-139-5p were inversely correlated with BCL2 expression, which suggested that miR-139-5p in colorectal cancer acted as a strong tumour suppressor by regulating the invasiveness and drug resistance. miR-139-5p was also confirmed to be a tumour suppressor of other cancers, including gastric carcinoma^20^, breast carcinoma^21^, lung cancer^22^, and hepatocellular carcinoma^23^,^24^.

EMT is the process by which epithelial cells are transformed into mesenchymal cells^25^. EMT may be physiological, as part of embryological development, or pathological, as part of cancer development, and is one of the key initiating events in the metastatic cascade. EMT has profound effects on tumour cell invasiveness, proliferation and motility and has also been shown to be an important step in inducing drug resistance in cancer cells against conventional therapeutics^26^,^27^,^28^. Researchers have demonstrated that alterations in the expression of critical molecules have been observed during the acquisition of the EMT phenotype^29^,^30^. In our study, it was confirmed that miR-139-5p can not only inhibit EMT but can also enhance drug resistance of 5-FU and OXA in colorectal cancer.

We identified BCL2 as a direct target of miR-139-5p in colorectal cancer cells and showed that the tumour suppressor activity of miR-139-5p is mediated by the modulation of BCL2 expression. BCL2 family proteins regulate and contribute to programmed cell death or apoptosis. The cell apoptosis results showed the induction of apoptotic cells contributed greatly to 5-Fu and OXA drug sensitivity, which was consistent with the multidrug resistance mechanisms. Our study provides new insight into the mechanism of miR-139-5p-mediated drug resistance and apoptosis that is different from a previously published article^22^. Defects in cell death signalling are a hallmark of cancer, particularly apoptotic cell death, which is often inhibited in tumour cells due to overexpression of anti-apoptotic proteins or decreased expression of proapoptotic proteins. For example, the BCL2 gene is overexpressed in many solid tumour cell lines, contributing to resistance to chemotherapy and radiotherapy^17^. Furthermore, downregulation of BCL2 sensitizes cells to chemotherapy^31^. BCL2 also serves as a regulator of cancer cell invasion and metastasis^32^. Recently, it was reported that BCL2 physically interacts with Twist1, which leads to transcriptional regulation of the Twist target genes, such as E-cadherin^14^,^15^. Thus, BCL2 may be directly involved in transcriptional regulation of the EMT process. These results support our current findings and suggest a potential mechanism for the tumour suppressor role of miR-139-5p mediated by the downregulation of BCL2.

In conclusion, the present study showed that miR-139-5p is downregulated in colorectal cancer cells and tissues, and its inhibitory effects on cell migration, invasion, and drug sensitivity are mediated by the downregulation of its target BCL2. The present results elucidate a potential mechanism underlying the tumour-suppressor role of miR-139-5p and indicate that miR-139-5p could be a useful marker and potential therapeutic target in colorectal cancer.

## Materials and Methods

### Study population

Human colorectal cancer samples were collected from 204 patients after surgical resection at Fudan University Shanghai Cancer Center, Shanghai, China, from January 2009 to December 2010. Fifty-four normal colorectal tissues were used as the control. Written informed consent was obtained from the patients, in accordance with the institutional guidelines, before sample collection, and the study was approved by the Committees for the Ethical Review of Research at the Fudan University Shanghai Cancer Center. The methods were performed in accordance with the approved guidelines. All patients had a histological diagnosis of colorectal cancer and received radical resection. None of the patients included in the study had received neoadjuvant therapy before surgery. The matching adjacent noncancerous tissue and primary colorectal cancer tissue were retrieved from the Tissue Bank of Fudan University Shanghai Cancer Center.

### Cell culture

Human colon cancer cell lines HT-29, HCT116, SW480, SW620, RKO, COLO205, Ls174T and LoVo were originally purchased from the American Type Culture Collection (ATCC). The cells were cultured in RPMI 1640 medium supplemented with 10% foetal bovine serum. Cells were cultured in a humidified 37 °C incubator supplemented with 5% CO2.

### RNA and miRNA extraction and quantitative real-time polymerase chain reaction

Total RNA was isolated from cultured cells and human tissues (204 cancer tissues and 54 normal colorectal tissues) using Trizol reagent. First strand cDNA was synthesized using the PrimeScriptTM RT Master Mix (Perfect Real Time) Kit (RR036A, Takara, China), which was then used for real-time polymerase chain reaction (PCR), together with forward and reverse primers and the Power SYBR Green PCR Master Mix (Life Technology, USA). GAPDH was used as an internal control for the BCL2 transcript levels. The primer sequences were as follows: BCL2-forward: CTGGTGGGAGCTTGCATCAC, reverse: ACAGCCTGCAGCTTTGTTTC; GAPDH-forward: GCACCGTCAAGGCTGAGAAC, reverse: ATGGTGGTGAAGACGCCAGT.

miRNA from tissues and cells was extracted according to the manufacturer’s instructions using a miRVANA Kit (Ambion, Carlsbad, MA), and the expression levels of miRNA-139-5p were detected by the Power SYBR Green PCR Master Mix, using U6 small nuclear RNA as an internal control. The relative expression of target genes was determined using the 2^−ΔΔCt^ method^33^.

### Vector construction, lentivirus production, and cell transfection

Lentiviral vectors that overexpressed miR-139-5p (pCDH-CMV-miR-139-5p) were purchased from RiboBio (Guangzhou, China). An empty lentiviral vector served as the control. A BCL2 restoration vector was constructed by introducing the BCL2 coding sequence, which was amplified from RKO cDNA using the primer pair forward: 5′-ATAGCTAGCGCCACCATGGCGCACGCTGGGAGAACA-3′ and reverse: 5′-ATAGCGGCCGCTCACTTGTGGCCCAGATAGGCAC-3′ into the pCDH-CMV-EF1-Puro vector. The wild-type (WT) 3′-UTRs of BCL2 containing putative miR-139-5p binding sites were isolated by PCR using the primer pair forward: 5′-ATACTCGAGAGTCAACATGCCTGCCCCAAACA-3′ and reverse: 5′-CACGCGGCCGCCTAGACAGACAAGGAAAGTTTAATGG-3′. Mutant (MUT) BCL2 3′-UTRs were generated by point mutation using a Quick-Change Site-Directed mutagenesis kit (Stratagene, La Jolla, CA) using the primer pair forward: 5′-ATTACGACATGTATCAATGCATAGGGAAGGAA-3′ and reverse: 5′- AAGGGTCGTGGCTCCCATGCTCCACGTGAAA-3′. WT and MUT BCL2 3′-UTRs were cloned into the psiCheck2 vector (Promega, Madison, WI). All of the constructed vectors were verified by sequencing.

### Lentivirus production and infection

pCDH-CMV-miR-139-5p mixed with the packaging plasmids psPAX2 and PMD2.G was co-transfected into HEK293T cells using Lipofectamine 3000 reagent (Invitrogen, USA) according to the manufacturer’s protocol. Virus particles were harvested 48 h after transfection. HCT116 and SW620 cells were infected with the harvested recombinant lentivirus and were isolated using puromycin to establish stable cell lines constitutively expressing miR-139-5p.

### Cell proliferation assay

Cell proliferation was assessed by the Cell Counting Kit-8 (CCK-8, Dojindo) assay as previously described^34^. Briefly, 2,000 cells were plated in 96-well plates, incubated and detected with the CCK-8 according to the manufacturer’s instructions.

### Cell migration and invasion assay

The migration ability of HCT116 and SW620 cells was tested in a Transwell Boyden Chamber (8-mm pore size, BD Biosciences), as previously described^34^. For the cell invasion assay, the polycarbonate membranes of the upper compartment of the chambers were precoated with a matrix gel.

### Assessment of chemotherapy sensitivity and apoptosis

HCT116 and SW620 cells stably expressing miR-139-5p or the control vector were treated with 5-FU (range, 0–1904 μM) or oxaliplatin (range, 0–2000 μg/μL), and cell inhibition was assessed by CCK-8 assay. The half maximal inhibitory concentration (IC50) was calculated. For the apoptosis analysis, colorectal cancer cells were treated with 1000 μM or 1 mg/mL oxaliplatin for 48 hours. The cells were then harvested and subjected to apoptosis analysis using an Annexin V-FITC and propidium iodide labelling kit (Invitrogen).

### Luciferase reporter assay

HCT116 cells were cultured in 96-well plates and cotransfected with 50 nmol/L of miR-139-5p mimic (or NC), 50 ng of luciferase reporter, and 10 ng of pRL-CMV Renilla luciferase reporter using Lipofectamine 3000. Forty-eight hours after transfection, the luciferase activities were assayed using a luciferase assay kit (Promega).

### Western blot analysis

Protein was extracted, separated by 10% SDS polyacrylamide gel electrophoresis and transferred to PVDF membranes. Membranes were blocked with 5% non-fat dry milk in TBST and were incubated with the following primary antibodies: anti-BCL2 antibody (1:1000, Epitomics, USA), anti-GAPDH (1:1000, Santa Cruz Biotechnology, CA), anti-E-cadherin, Vimentin, ZEB1 rabbit polyclonal antibody (1:1000 dilution; Abcam, USA), anti-β-actin mouse polyclonal antibody (1:1000 dilution; CST, USA). Secondary antibodies were added, and the bands were visualized using the enhanced chemiluminescence (Pierce, Thermo Scientific, USA). GAPDH was used as the loading control.

### Confocal immunofluorescent analysis

Cells (5 × 10^4^) were implanted onto a slide for 24 hours. The cells were fixed with paraformaldehyde for 30 minutes, permeabilized with 0.1% Triton X-100 for 5 min at room temperature and the primary antibodies were added incubation for 2 hours at room temperature. Then, the cells were incubated with Alexa Fluor 594 TgG donkey anti-rabbit (1:500, Invitrogen, USA) and Alexa Fluor 488 TgG goat anti-mouse (1:500, Invitrogen, USA) for an hour at room temperature. Nuclei were stained with propidium iodide for 5 minutes when necessary. Fluorescence images were photographed with a confocal microscope.

### Mouse model

MiR-139-5p knockout (KO) and littermate controls, all on C57BL/6, were originally purchased from the Genetically Modified Animal Center (East China Normal University at Shanghai). The miR-139-5p gene was knocked out by CRISPR/Cas9 in the whole body, as described previously^35^. Briefly, we first designed the gDNA primer pair (sense: GGAGGCAACTGAAGATGGGTA; anti-sense: GGAGGCATAAGGGTGAGAAAG), and constructed guide RNA and a Cas9 transcription template. Then, we performed Cas9 and gRNA *in vitro* transcription and microinjection. Finally, the target region of the genomic DNA was amplified and detected. miR-139-5p KO mice were further backcrossed to C57BL/6 background for 10 generations. All mice were 6–8 weeks old and were bred in-house to generate comparable groups.

Colitis-associated tumourigenesis was conducted following a previously published protocol^35^,^36^. Briefly, mice were injected intraperitoneally with 10 mg/kg azoxymethane (AOM, Sigma–Aldrich), and five days later, the mice were treated with 2.5% dextran sulphate sodium (DSS) in their drinking water for 7 days, followed by 14 days of regular water. The cycles was repeated two additional times, and mice were sacrificed at the end of the DSS cycle. Body weight was recorded daily. Mice were sacrificed at the indicated time intervals, and the number of tumours and size were measured in a blinded fashion.

All proposals were approved and supervised by the institutional animal care and use committee of Fudan University. All animal studies were conducted in accordance with the National Institute of Health guidelines for the Care and Use of Laboratory Animals.

### Histological analysis

Paraffin-embedded colorectal tissues were longitudinally cut into 4 μm sections and were stained with haematoxylin and eosin solution (H&E). Immunohistochemistry (IHC) staining was performed as described previously^34^,^36^. BCL2 anti-human rabbit antibody was used at a dilution of 1:100 (Epitomics, USA); PBS was used as a negative control. Each section was evaluated and scored independently by two pathologists. A semiquantitative scoring system was used in this trial^34^,^37^.

### Statistical analysis

SPSS 22.0 software was used for the statistical analysis. Data were expressed as the mean** ± **SD. Comparisons between two independent groups were performed by Student’s t-test. Spearman’s correlation analyses were used to identify the correlation between miR-139-5p and BCL2. P values < 0.05 were considered statistically significant.

## Additional Information

**How to cite this article**: Li, Q. *et al.* miR-139-5p Inhibits Epithelial-Mesenchymal Transition and Enhances the Chemotherapeutic Sensitivity of Colorectal Cancer Cells by Downregulating BCL2. *Sci. Rep.* **6**, 27157; doi: 10.1038/srep27157 (2016).
